# Role of JMJD6 in Breast Tumourigenesis

**DOI:** 10.1371/journal.pone.0126181

**Published:** 2015-05-07

**Authors:** Coralie Poulard, Juliette Rambaud, Emilie Lavergne, Julien Jacquemetton, Jack-Michel Renoir, Olivier Trédan, Sylvie Chabaud, Isabelle Treilleux, Laura Corbo, Muriel Le Romancer

**Affiliations:** 1 Université de Lyon, F-69000 Lyon, France; 2 Université Lyon 1, F-69000 Lyon, France; 3 Inserm U1052, Centre de Recherche en Cancérologie de Lyon, F-69000 Lyon, France; 4 CNRS UMR5286, Centre de Recherche en Cancérologie de Lyon, F-69000 Lyon, France; 5 Equipe Labellisée "La Ligue; 6 Centre Léon Bérard, Biostatistics Unit, F-69000 Lyon, France; 7 UMR CNRS 8203 Vectorology and anti-cancer therapeutics, Institut Gustave Roussy, 114, Rue E. Vaillant, 94805 Villejuif Cedex, France; 8 Centre Léon Bérard, Department of Medical Oncology, F-69000 Lyon, France; 9 Centre Léon Bérard, Pathology Department, F-69000 Lyon, France; II Università di Napoli, ITALY

## Abstract

**Background:**

Protein arginine methylation is a common post translational modification that regulates protein properties. This modification is carried out by a family of nine arginine methyltransferases (PRMTs). Arginine methylation has already been linked to tumourigenesis as overexpression of these enzymes was associated with various cancers, notably in breast cancers. Since the Jumonji Domain Containing 6 protein (JMJD6) possesses an arginine demethylase activity able to remove the methyl mark, we wanted to assess its potential role in breast tumourigenesis.

**Methods:**

The expression of the protein by tissue microarray immunohistochemical staining was performed on a cohort of 133 breast tumours. Using cell lines stably overexpressing or knocked down for JMJD6, we evaluated its role on cell proliferation, cell migration, colony formation and mice tumour xenografts.

**Results:**

The analysis of JMJD6 expression in a cohort of breast tumour samples indicates that JMJD6 was highly expressed in aggressive breast tumours. Moreover, high expression of JMJD6 was associated with poor disease-free survival of patients in this cohort. JMJD6 silencing in breast tumoural cells promotes certain characteristics of tumourigenesis including proliferation, migration *in vitro*, and tumour growth *in vivo*. These effects are dependent on its demethylase activity as an enzymatic dead mutant lost these properties.

**Conclusions:**

Although JMJD6 displays anti-tumoral properties in cell lines, its expression in breast tumours may be a marker of poor prognosis, suggesting that its function could be altered in breast cancer.

## Introduction

In mammalian cells, methylation of arginine is performed by a family of protein arginine methyltransferase (PRMT) catalysing mono or dimethylation. They are classified as Type I or Type II based on their ability to respectively dimethylate asymmetrical (ADMA) or symmetrical (SDMA) arginine residues [1]. Although arginine methylation has been shown to be extensively involved in signal transduction [2–4], gene transcription [5,6] and mRNA splicing [7,8], it is only recently that this modification has been linked to carcinogenesis (For a review, [9]) Several studies reported that PRMTs expression is elevated in various types of human cancers including breast cancers [9] and silencing of PRMTs in breast cancer cell lines slows proliferation [4,10,11]. It has also been shown that an increasing number of proteins contributing to tumourigenesis are methylated on arginine including p53 [12], ERα [4], BRCA1 [13], EGFR and the tumour suppressor protein programmed cell death protein 4 (PDCD4) [14]. Moreover, ERα has been shown to be hypermethylated in aggressive breast tumour samples [15], supporting the idea that arginine methylation might be involved in breast tumourigenesis.

Although arginine methylation was initially described as a stable mark, several studies have identified a number of proteins in which methylation is dynamic [4,16]. These results supported the existence of an arginine demethylase capable of removing the methyl marks. The first arginine, demethylase Jumonji C domain-containing protein 6 (JMJD6), was identified by the Bruick laboratory [17], who reported that JMJD6 was capable of demethylating histone H3 at arginine 2 (H3R2) and histone H4 at arginine 3 (H4R3). Thereafter, several reports have described that JMJD6 also acts as a lysine hydroxylase [18–20] modifying, for instance, the transcriptional activity of p53 [21]. In addition, Rosenfeld’s team confirmed that JMJD6 possesses a demethylase activity both on histone and non-coding RNA [22] and our team has recently shown that JMJD6 demethylates a non-histone substrate, the oestrogen receptor alpha, in which methylation regulates the non-genomic signalling of oestrogens [23].

As arginine methylation is known to be involved in tumourigenesis, we wanted to assess the involvement of the arginine demethylase JMJD6 in breast cancer.

## Materials and Methods

### Plasmids

pCDNA3-JMJD6-V5 wild type and mutant catalytically inactive (H187A, D189A, H273A) were gifts from Richard Bruick [17]

### Cell Culture

MCF-7 mammary cells were maintained at 37°C in DMEM (Dulbecco’s Modified Eagle’s Medium) supplemented with 10% foetal calf serum and 1% non-essential amino acids. MCF-7 cells have been certified by CelluloNet, Lyon, France.

### Generation of stable cell lines

MCF-7 cell line was transfected with 5μg of vectors expressing JMJD6 WT (pcDNA3-JMJD6WT-V5), JMJD6 mut (pCDNA3-JMJD6MUT-V5) or with empty pCDNA3 (2 X 10^6^ cells in a 100mm dish) using Exgen 500 (Euromedex) according to the manufacturer’s guidelines. Selection was initiated 48h after transfection in medium containing 750μg/ml of G418. After selection, cells were maintained in medium containing 300μg/ml of G418. Three commercially available lentiviral short hairpin RNA (shRNA) constructs targeting human JMJD6 and one negative control construct created in the same vector backbone (pLKO.1-Puro) were purchased from SIGMA. Puromycin selection (1μg/mL) was started 48h after lentiviral infection. The established cell lines have been already described and characterized [23].

### Western blotting

Cells were lysed in RIPA buffer containing 50mM Tris HCl, pH 8, 150mM NaCl, 1mM EDTA, 1% NP-40, 0.25% deoxycholate. The cell lysates were separated on SDS-PAGE. The proteins were visualised by an enhanced chemiluminescence kit (Roche Molecular Biochemicals).

### Proliferation assay

Cells were plated in triplicate in 96-well plates at a density of 1000 cells per well. One plate was prepared each day of the time course. At each time point, cells were treated with Uptiblue (Interchim) and incubated 3h at 37°C. Fluorescence intensity was monitored at 530–560 nm excitation wave length and 595nm emission wave length (CytoFluor, PerSeptive, Biosystem). Each experiment was performed in triplicate.

### Wound-healing assay

Cells were plated in duplicate in 6-well plates and grown to confluence. Then, wounds were performed with a p200 pipette tip. After washes to remove cellular debris, three images of each well were taken. The width of the wound was measured at 3 places and corded as t = 0. The cells were then allowed to migrate back into the wounded area. Sixteen hours later, the width of the open area was measured. Cell migration was expressed as the percentage of the gap across the gap (t = 16) relative to the primary width of the open area (t = 0). Images were acquired on a phase contrast microscope (Zeiss, Axiovert 25). All experiments were performed in triplicate.

### Colony formation assay

5000 cells were mixed in Top Agar (1X medium, 0.45% agar) on 6-well plates containing 1.5ml of Base Agar (1X medium, 0.75% agar). Plates were incubated at 37°C for 3 weeks. Colonies were fixed with methanol and stained with 6% glutaraldehyde and 0.5% crystal violet. Microscopic colonies composed of more than 50 cells were counted in each well. Experiments were performed in triplicate.

### Xenograft studies

10 female nude mice (6–8 weeks old) were purchased from Harlan Laboratories, maintained and treated under specific pathogen-free conditions. The Institutional Animal Care and Use Committee of evaluation of Lyon (Comité d’Evaluation Commun au PBES, à AniCan, au laboratoire P4, à l’animalerie de transit de l’ENS, à l’animalerie de l’IGFL, au PRECI, à l’animalerie du Cours Albert Thomas, au CARRTEL INRA Thonon-les-Bains et à l'animalerie de transit de l'IBCP, CECCAPP, number C2EA) approved this study that is conforming to the federal guidelines for the care and maintenance of laboratory animals. The mice were injected subcutaneously with 5 X 10^6^ of MCF-7 cells shCtr (5 mice) or with shJMJD6 (5 mice) resuspended in Matrigel into the mammary gland. Access to food and water was unrestricted. Oestrogen solution (10mM) was brushed twice a week to the neck. Tumour formation was measured with a caliper every 2 to 3 days for the duration of the experiment. At the end of the study, mice were sacrificed by cervical dislocation. Tumours were harvested, photographed, measured and weighed. We did not use any anesthesia or analgesia.

### Human breast tissue samples collection

JMJD6 expression was analysed on 3 human normal breast samples from mammoplasties and tumours from 133 patients treated in our institution (Centre Léon Bérard) with invasive non-metastatic breast cancer and whose clinical and biological data were available from the regularly updated institutional database. Written informed consent was obtained from each patient. The study protocol was approved by the institutional ethics committee of Centre Léon Bérard in Lyon. Patients’ characteristics are presented in S1 Table. Median age was 57 (range 32–87) and the majority of patients were post-menopausal (65%). In our study, the classification of ERα and PR negative tumours were respectively determined by tumours exhibiting less than 10% of receptor positive cells. JMJD6 expression was evaluated both in terms of percentage of stained cells and intensity (S2 Table), but only results concerning percentage of stained cells are presented here.

### Immunohistochemistry

Paraffin embedded tumours tissue fixed in formalin were used for analysis. The pathologist selected representative areas from breast invasive carcinomas. Triplicates from each tumour were inserted into a tissue microarray (TMA) block which contained 40 tumours. Four TMA (160 tumours) were analysed. The blocks containing invasive carcinoma were serially sectioned at a thickness of 4μm. After deparaffinisation and rehydration, tissue sections were boiled in 10mM citrate buffer pH 9 using a water bath at 97°C for 40 minutes. To block endogenous peroxidases, the slides were incubated in 5% hydrogen peroxide in sterile water. The slides were then incubated at room temperature for 1 hour with the anti-JMJD6 monoclonal mouse antibody (sc-28348, Santa Cruz, 1/100). After rinsing in Phosphate Buffer Saline, the slides were incubated with a biotinylated secondary antibody bound to a streptavidin peroxidase conjugate (Envision Flex kit Ref: K800021-2, Dako, Trappes, France). Bound antibody was revealed by adding the substrate 3,3-diamino benzidine. Sections were counterstained with hematoxylin. Immunohistochemistry results were assessed by two pathologists in a blind study, analysing the percentage and intensity of nuclear staining in tumoural cells.

### Statistical analysis

#### Descriptive analysis

Distribution of clinical parameters (clinical, histological and immunohistochemical data) was compared between JMJD6 expression groups, using Pearson’s χ^2^ test or Fisher’s exact test.

#### Survival analysis

Overall Survival (OS) defined as time from diagnosis to death or date of last follow-up and Disease Free Survival (DFS) defined as time from diagnosis to relapse, death or date of last follow-up (for censored patients) were studied.

Survival distributions were estimated by Kaplan-Meier method and compared between expression level groups using the Log-Rank test. To evaluate a possible relationship between DFS and JMJD6 expression, univariate Cox proportional hazard regression models were built by considering JMJD6 expression and some covariates, approved to be prognostic of DFS (tumour size, lymph node involvement, ERα, PR, HER2 status and SBR grade). All variables significant at 10% in univariate analysis were included in the initial multivariate model, as well as interactions between them, significant at 5% level. A backward manual selection procedure was used to lead to the final model by removing non-significant variables (p>0.05).

All statistical analyses were performed using SAS software, v 9.3 (SAS institute Inc, Cary, NC, USA).

## Results

### JMJD6, a marker of bad prognosis in breast cancer

**Fig 1:**
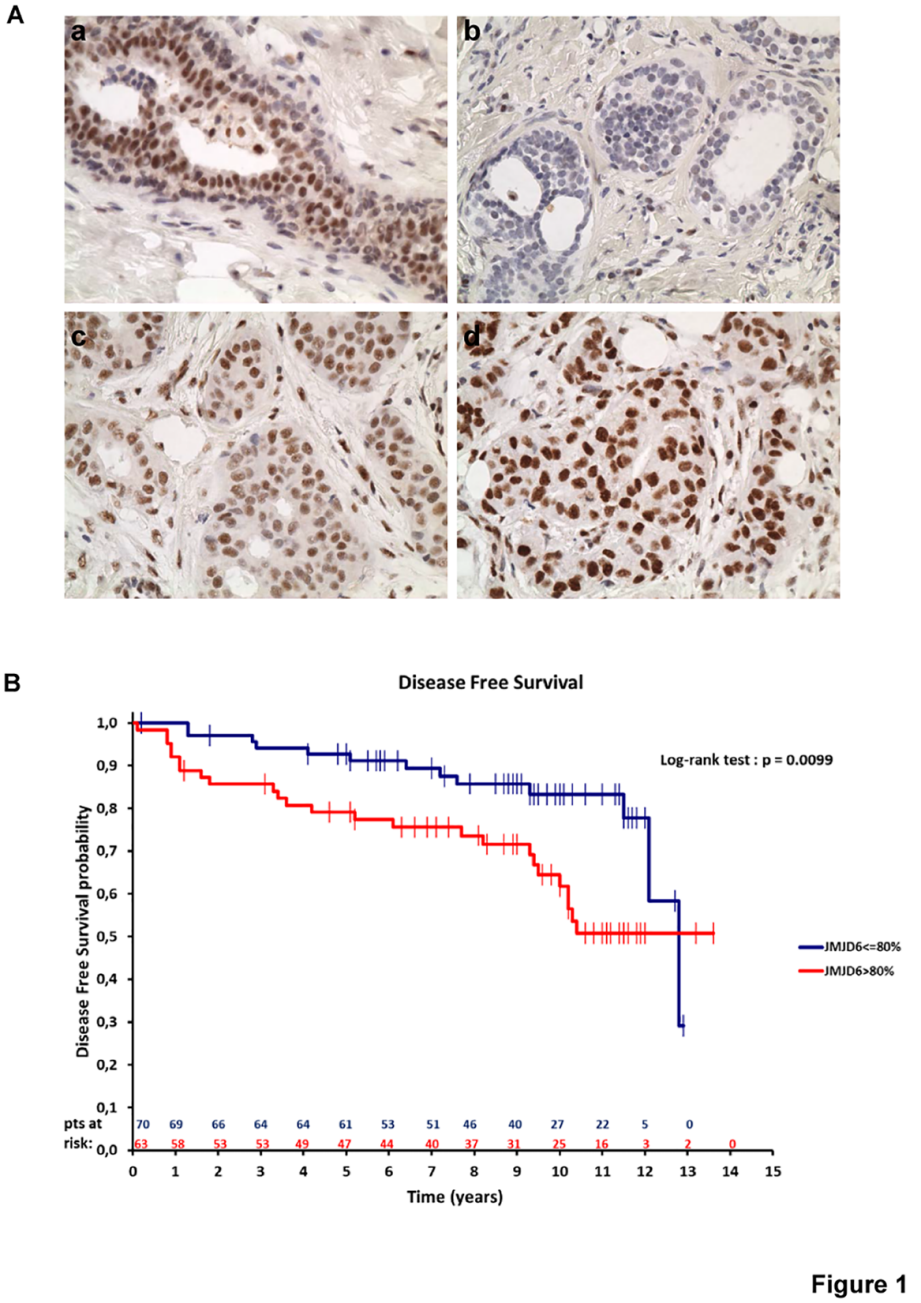
JMJD6 expression in breast tumours. **(A)** JMJD6 expression has been analysed by IHC in normal mammary gland (panel a) as well as in infiltrating breast cancer showing low staining (panel b) intermediate (panel c) or strong staining (panel d) (objective X 40). **(B)** Kaplan Meier estimates of DFS according to JMJD6 expression groups.

To investigate the role of JMJD6 in breast carcinogenesis we examined JMJD6 expression in normal and tumoural human breast (Fig 1A). Immunohistochemical analysis revealed that in normal breast, JMJD6 expression was restricted to the nuclei of epithelial cells whereas myoepithelial cells were not stained (Fig 1A, panel a). Tissue microarray analysis of a cohort of 133 breast cancer samples by IHC confirmed the nuclear localisation of the protein. Interestingly, the nuclear staining varied from low level (Fig 1A, panel b) to intermediate (panel c) and strong (panel d).The distribution of JMJD6 expression among the 133 breast tumours is presented in S1 Table. In each case, the percentage and the intensity of stained tumour cells were analysed separately and cut-off points were chosen to obtain comparable sub-group size, in order to ensure the best statistical power.

In terms of patient outcome, according to univariate analysis, high JMJD6 expression (>80% of stained cells) was significantly associated with poor disease-free survival (DFS, p = 0.0099) (Fig 1B) although no significant association was found with overall survival (OS) (S1 Fig). Multivariate Cox analyses revealed that a high expression of JMJD6 remained an independent poor prognostic factor for DFS (Table 1).

**Table 1:** Multivariate analysis of DFS integrating JMJD6 expression.

Variable	HR	95% CI	p-value
**JMJD6: % of stained cells**			
**< = 80%**	**1**	**[.-.]**	**0.0306**
**> 80%**	**2.11**	**[1.07–4.15]**	
Tumor size (mm)			
< 30 mm	1	[.-.]	0.0034
> = 30 mm	2.62	[1.38–4.98]	

Final multivariate Cox modelisation of DFS adjusted on JMJD6 expression. Hazard ratio for high JMJD6 expression (>80% stained cells) relative to low JMJD6 expression (<80% stained cells) is presented, adjusted on tumour size.

The association between JMJD6 expression and clinical parameters showed that a high expression of JMJD6 was significantly associated with age <50 years and non-menopausal status with (respectively 42.9% and 43.5% versus 25.7% and 27.5% of patients with a lower expression of JMJD6, p = 0.037 and p = 0.055). High expression of JMJD6 was also associated with higher SBR grade (46.0% of patients with a high expression of JMJD6 had SBR grade 3 vs. 22.9% of patients with a low expression of JMJD6, p = 0.002) (Table 2). Moreover, high expression of JMJD6 was associated with non-luminal A breast tumours (54.1% versus 27.5% of patients with a low expression of JMJD6, p = 0.002).

**Table 2:** Distribution of clinical parameters according to groups to JMJD6 expression.

	JMJD6: % of stained cells				
	< = 80% N = 70		> 80% N = 63		p-value
	n	%	n	%	
**Age at diagnosis (years)**					**χ** ^**2**^
< 50	18	(25.7%)	27	(42.9%)	**p = 0.037**
> = 50	52	(74.3%)	36	(57.1%)	
**Menopause**					**χ** ^**2**^
ND	1		1		**p = 0.055**
No	19	(27.5%)	27	(43.5%)	
Yes	50	(72.5%)	35	(56.5%)	
**Tumor size (mm)**					**χ** ^**2**^
< 30 mm	54	(77.1%)	42	(66.7%)	p = 0.178
> = 30 mm	16	(22.9%)	21	(33.3%)	
**Histological grade (SBR)**					**χ** ^**2**^
1	23	(32.9%)	7	(11.1%)	**p = 0.002**
2	31	(44.3%)	27	(42.9%)	
3	16	(22.9%)	29	(46.0%)	
**Lymph node involvement**					Fisher Exact
N0	34	(48.6%)	29	(46.0%)	p = 0.675
Micro metastasis	7	(10.0%)	4	(6.3%)	
Macro metastasis	29	(41.4%)	30	(47.6%)	
**Estrogen receptor**: % marked cells					**χ** ^**2**^
ND	1		0		p = 0.108
Negative	5	(7.2%)	11	(17.5%)	
Positive	64	(92.8%)	52	(82.5%)	
**Progesterone receptor**: % marked cells					**χ** ^**2**^
ND	1		0		p = 0.269
Negative	15	(21.7%)	19	(30.2%)	
Positive	54	(78.3%)	44	(69.8%)	
**Luminal A**					**χ** ^**2**^
ND	1		2		**p = 0.002**
No	19	(27.5%)	33	(54.1%)	
Yes	50	(72.5%)	28	(45.9%)	
**Phenotype**					Fisher exact
ND	1		2		**p = 0.015**
Luminal A	50	(72.5%)	28	(45.9%)	
Luminal B	14	(20.3%)	22	(36.1%)	
HER2+	1	(1.4%)	3	(4.9%)	
ER-/PR-/HER2-	4	(5.8%)	8	(13.1%)	

Clinical parameters (age at diagnosis, tumour size, menopausal status, lymph node involvement, SBR grading and hormonal receptor expression) were analysed for the 133 patients included in the TMA study. Association between clinical characteristics and the level of JMJD6 was determined using χ^2^ test or Fisher’s exact test. Significant correlations are highlighted in bold characters.

Altogether, these data show that a high expression of JMJD6 was associated with less favourable prognostic factors.

### JMJD6 controls proliferation and migration of breast cancer cells

**Fig 2:**
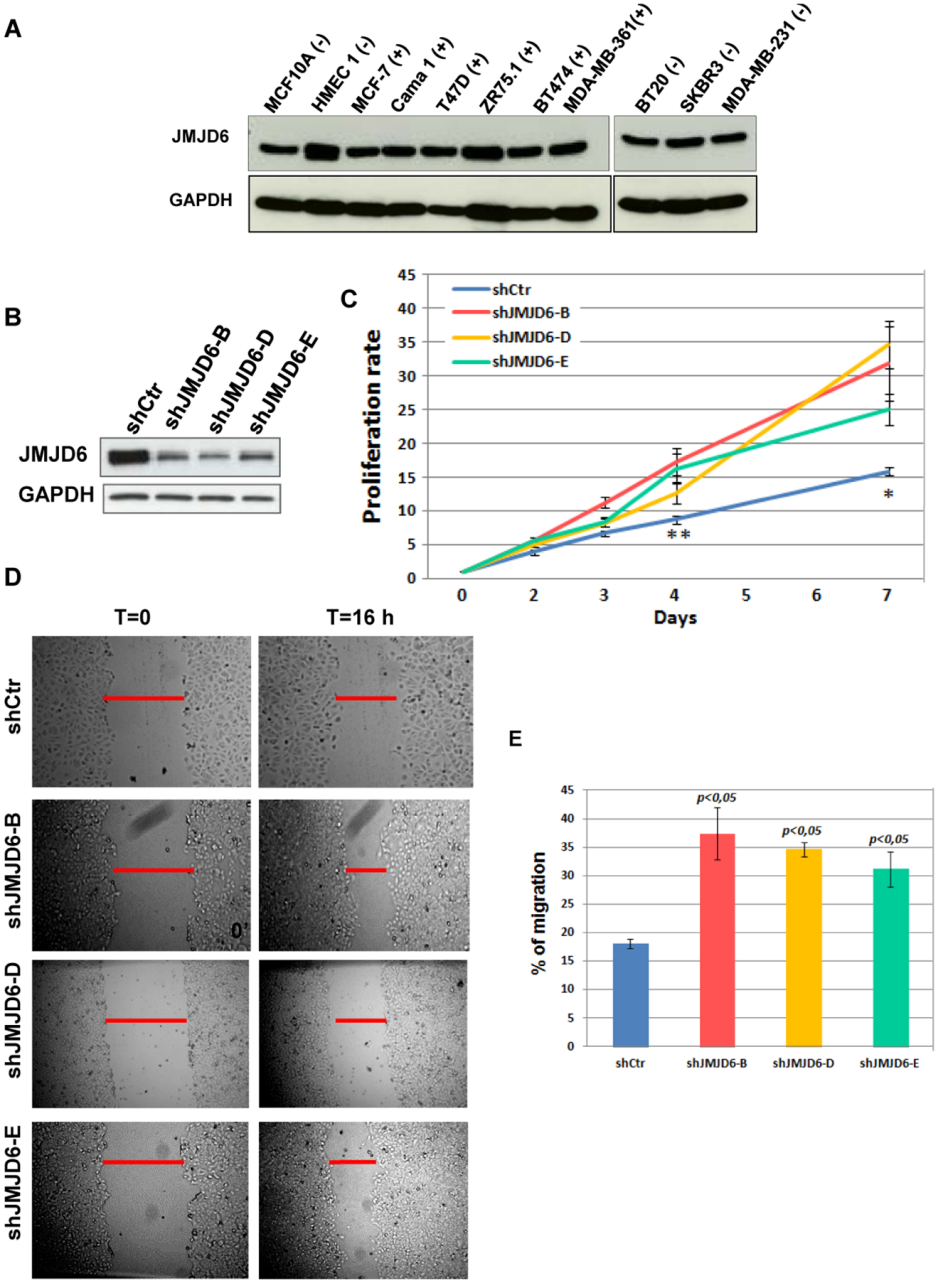
JMJD6 knock-down increases MCF-7 cell proliferation and migration. **(A)** JMJD6 expression has been studied in several human breast tumour cell lines by western blotting. GAPDH expression has also been determined to validate the quality of the samples loading. **(+)** and **(−)** indicates oestrogen receptor alpha expression in the different cell lines. **(B)** JMJD6 expression was analysed by western blotting to verify the efficiency of the ShRNAs. **(C)** Proliferation assays were performed on MCF-7 cells invalidated for JMJD6. Cell viability was measured for the indicated times after seeding. *p<0.05; **p<0.01. **(D)** Wound healing assays were performed in MCF-7 cells invalidated for JMJD6. **(E)** The percent of migration was determined as a mean of distances of the wound for the different experiments. Analysis was performed in three separate experiments. The data are represented as means +/- SEM from three replicates in each of the three independent experiments.

Based on our observations that high expression of JMJD6 associated with high histological grade and with aggressiveness of breast cancer, we hypothesised that JMJD6 might have a role in the regulation of growth and migration of breast cancer cells. In the first instance, we checked its expression in several human mammary cells including normal (HMEC1), transformed (MCF10A) as well as tumoural cells including both ERα-positive and ERα negative cells. As shown in Fig 2A, JMJD6 is highly expressed in all the analysed cell lines. Furthermore, its expression seems to be equivalent in normal and tumoural cells and independent of hormonal status. To assess the potential role of JMJD6 on breast cancer progression, we generated MCF-7 cell lines stably expressing shRNAs against JMJD6 (shJMJD6) or a control sequence (shCtr). Puromycin-resistant cells were characterised by western blot using an anti-JMJD6 antibody (Fig 2B). We analysed the effect of JMJD6 knock-down on cell proliferation and migration. The proliferation rate of these different cell lines was then measured by MTS assays. JMJD6 deprivation caused an increase in the proliferation rate of MCF-7 cells compared with the control cells (Fig 2C). Also, we found that JMJD6 deprivation increased cell migration of tumour cells analysed by wound scratch test (Fig 2D and 2E).

**Fig 3:**
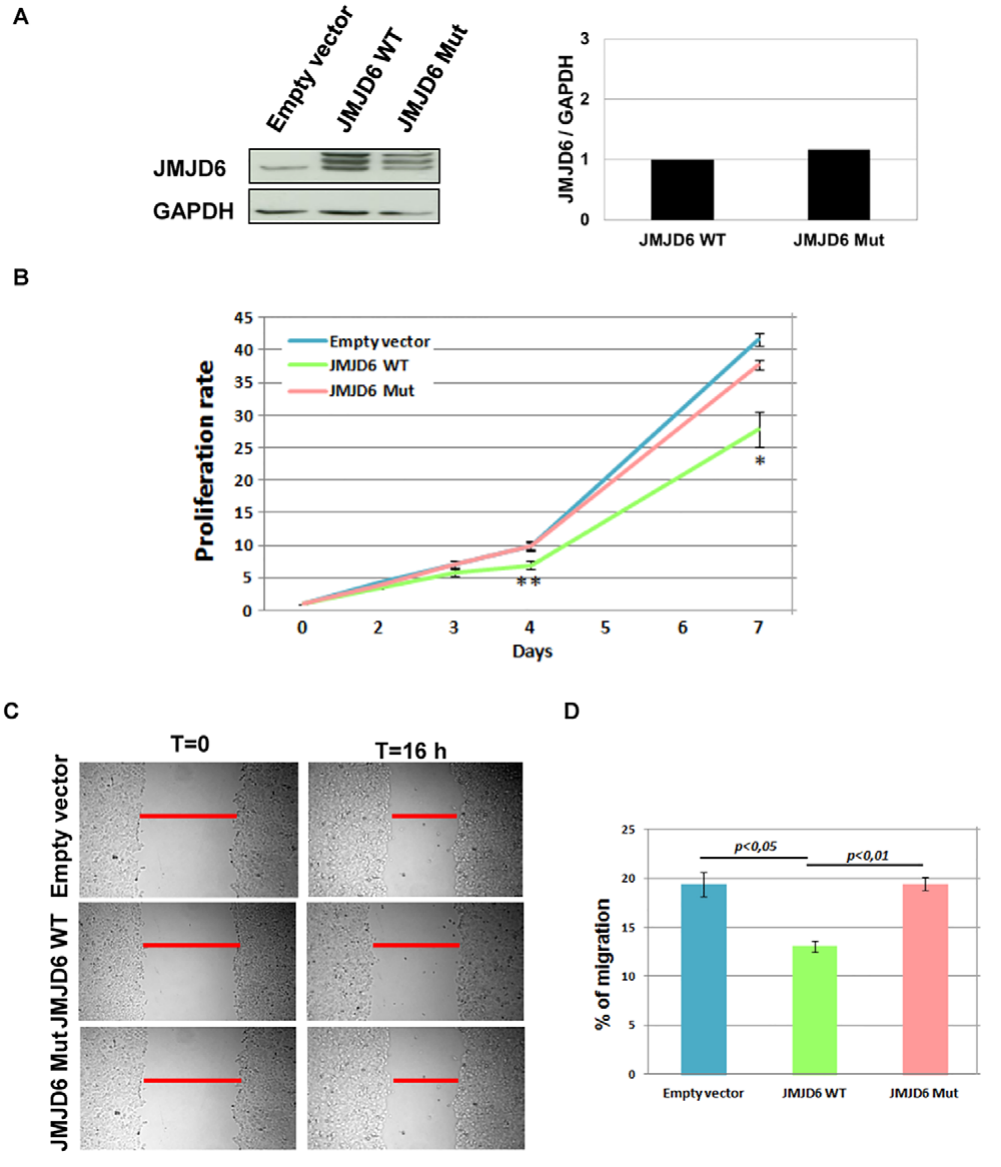
JMJD6 overexpression decreases MCF-7 cell proliferation and migration. **(A)**. JMJD6 expression was determined for MCF-7 stably expressing JMJD6 (WT or Mut). GAPDH serves as a control of loading. The lower band represents endogenous JMJD6 expression. The right panel shows the relative quantification of JMJD6/GAPDH expression. **(B)** Cell index growth was determined for MCF-7 stably expressing JMJD6 (WT or Mut) compared to the empty vector. *p<0.05; **p<0.01. **(C)** Wound healing assays were performed in MCF-7 cells stably expressing JMJD6 (WT or Mut). **(D)** The percent of migration was determined as in (Fig 2D). Analysis was performed in three independent experiments.

Conversely, when JMJD6 was stably overexpressed in MCF-7 cells we observed a significant reduction of proliferation as well as cell migration in these cells compared to the control (Fig 3B, 3C and 3D). Transfected proteins in neomycin-resistant cells were checked by western blot using an anti-JMJD6 antibody (Fig 3A). Interestingly, the enzymatically dead mutant (H187A, D189A, and H273A) did not affect MCF-7 cell proliferation and migration (Fig 3B, 3C and 3D), indicating that these effects depend on JMJD6 enzymatic activities.

### JMJD6 knock-down promotes tumourigenesis *in vitro* and *in vivo*

**Fig 4:**
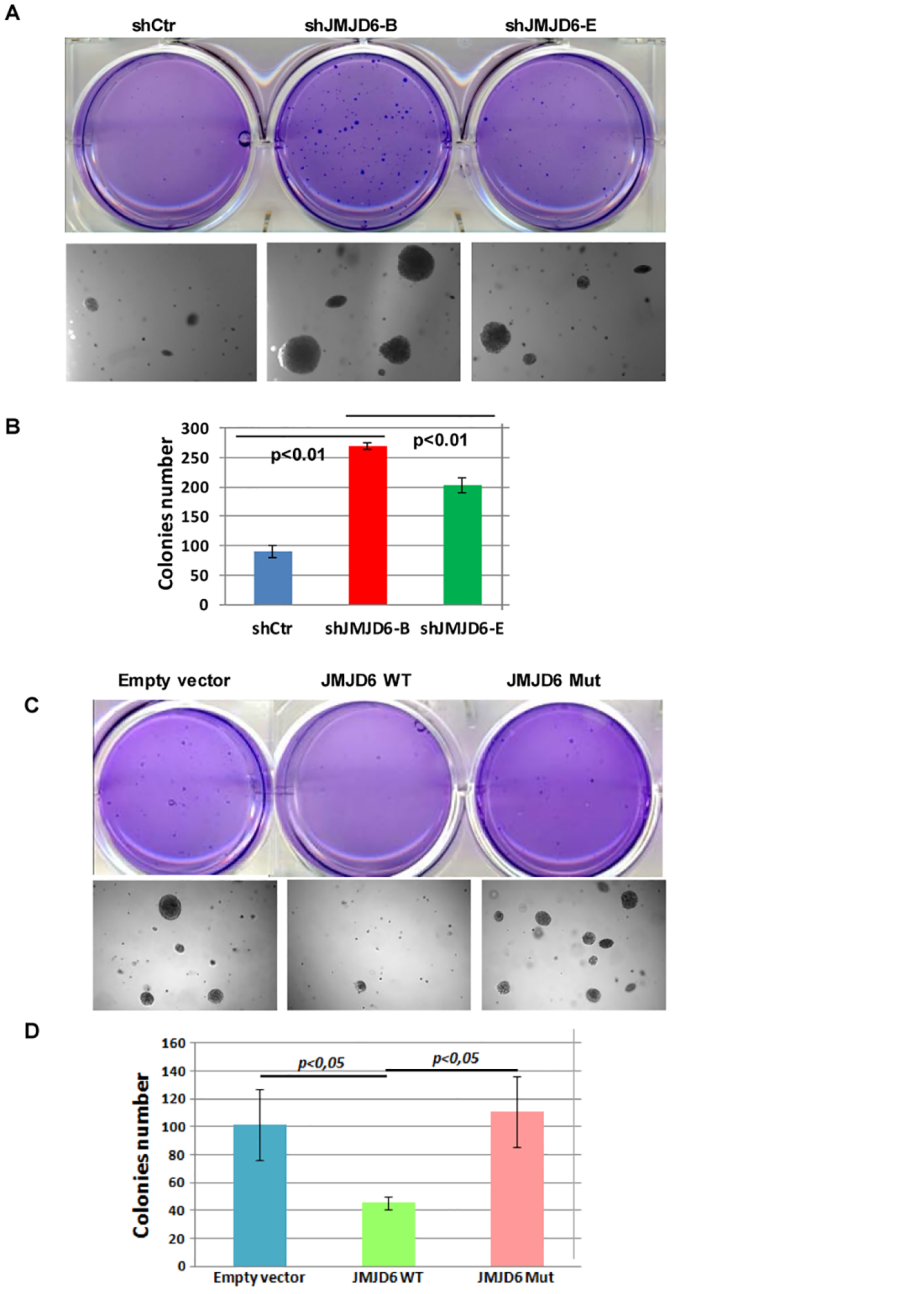
JMJD6 expression negatively affects MCF-7 anchorage-independent growth. **(A)** MCF-7 knocked down for JMJD6 were assessed for anchorage-independent growth 25 days after plating. The lower panel shows a magnification of the colonies. **(B)** Quantification of anchorage independent growth. Colonies composed of more than 50 cells were counted in each well. The data are represented as means +/- SEM from two replicates in each of the three independent experiments. **(C)** The same experiments were performed on MCF-7 cells stably expressing JMJD6 (WT or Mut). The lower panel shows a magnification of the colonies. **(D)** The quantification of colonies was performed as in (B). The data are represented as means +/- SEM from two replicates in each of the three independent experiments.

To determine whether JMJD6 depletion promotes an invasive phenotype, MCF-7 cells knocked down for JMJD6 were tested in an outgrowth assay, which is frequently employed as a reliable system to assess *in vitro* invasiveness of breast cancer cells. We found that shJMJD6 cells increased the number and the size of colonies (Fig 4A and 4B). Consistent with these results, overexpression of JMJD6 in MCF-7 cells clearly yielded fewer colonies than mock cells. Overexpression of JMJD6 mutant had no effect on anchorage-independent growth compared with the control cells (Fig 4C and 4D) confirming the involvement of JMJD6 enzymatic activities in breast cancerogenesis.

**Fig 5:**
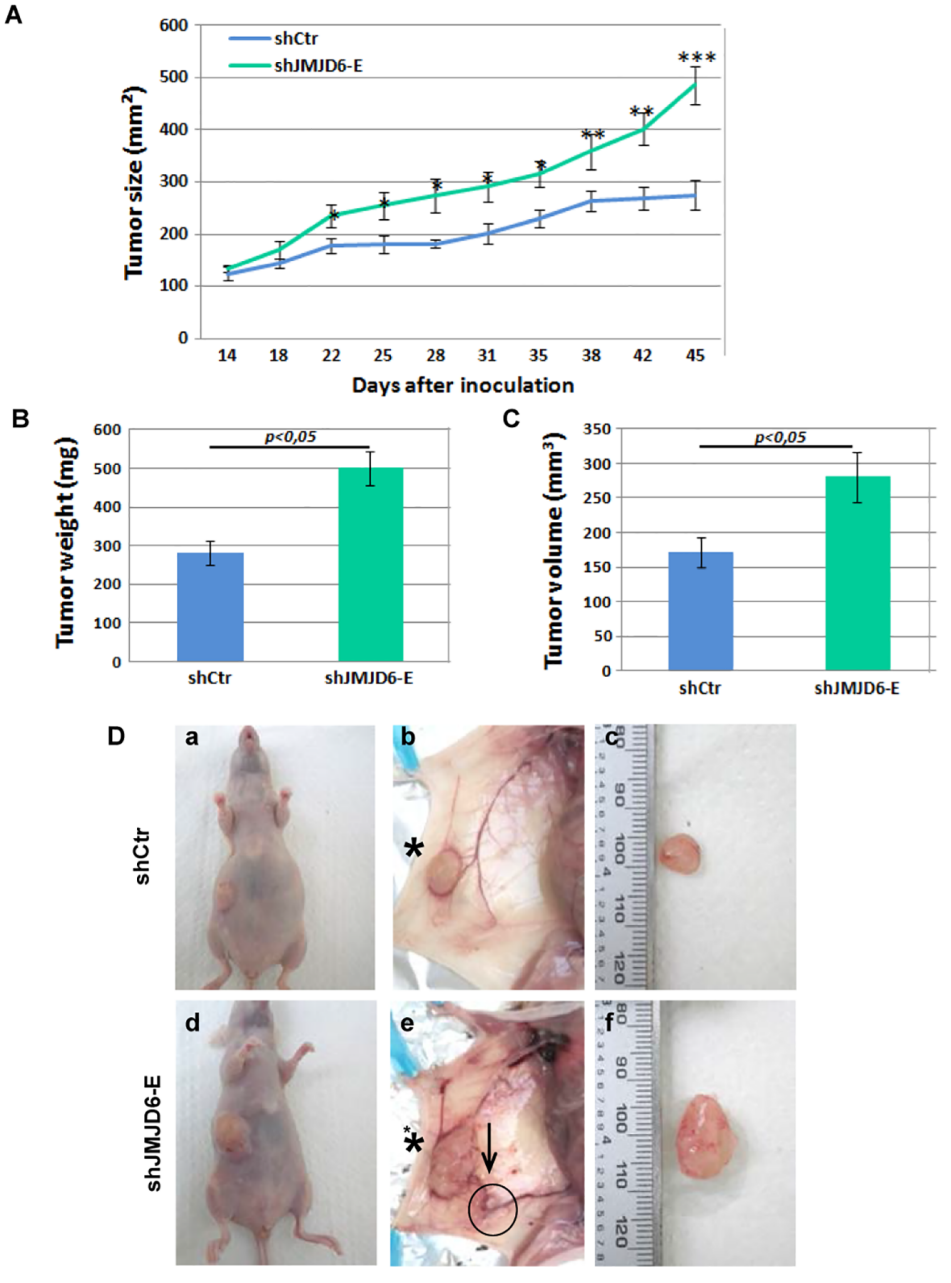
Knock-down of JMJD6 increases breast tumour xenograft growth. Orthotopic xenograft tumours were generated with MCF-7 breast tumour cells stably expressing either control shRNA or shRNA targeting the JMJD6 gene. **(A)** Tumour growth curve was performed measuring tumours twice per week with an electronic caliper. Values represent mean +/- SEM from 10 animals. * p<0.05, ** p<0.01 ***p<0.005 **(B)** Mice were dissected and tumours isolated at eight weeks following injection. Tumour mass **(B)** and volumes **(C)** were measured for each tumour. **(D)** Pictures of the tumours were taken from mice xenografted with MCF-7 control cells (a,b,c) and MCF-7 shJMJD6 cells (d,e,f). Panels a and d show the tumours in the intact mice, denoted by an asterisk. Panels b and e show the tumours before surgery. In panel e, the arrow indicates a secondary tumour. Panels c and f show the tumours removed after surgery.

Our results clearly indicate that JMJD6 possesses anti-tumoural properties. To investigate whether JMJD6 depletion accelerates tumorigenesis *in vivo*, we injected knocked down cells into female athymic nude mice. Forty-five days after inoculation, mice were sacrificed and tumours were weighed and measured. As shown in Fig 5A, tumours expressing shJMJD6 grew significantly faster than those in the control groups (p<0.005). Tumour volumes and weights were significantly higher in the mice injected with MCF-7/shJMJD6 compared to mice injected with MCF-7/shCTR (Fig 5B and 5C). Fig 5D shows an example of the tumours observed within the mice (panels a and d). Next, we observed the tumours after surgery and we found that a large proportion of the mice injected with MCF-7/shJMJD6 presented small tumours distant from the original ones (Fig 5D, panel e). This was observed in 8/10 tumours, although it was never observed with the control cells, suggesting that JMJD6 could be involved in tumour migration.

Altogether, these results clearly establish that JMJD6 possesses anti-tumoural properties as measured by its negative effect on cell proliferation, migration, colony formation and tumour engraftment. Moreover, these effects likely involved its enzymatic activities as the enzymatic dead mutant is unable to decrease breast tumourigenesis.

## Discussion

The JMJD6 protein was identified many years ago, but until recently its role was a matter of controversy. Formerly, JMJD6 was named the phosphatidylserine receptor (PTDSR), a cell surface protein that facilitates recruitment of phagocytic cells to sites of apoptosis [24]. Later, Cui et al found that PTDSR is also a nuclear protein with contains five nuclear localisation signals suggesting that it could have different functions [25]. More recently, PTDSR was renamed Jumonji domain containing 6 (*JMJD6*) based on the presence in its sequence of a JMJC domain found in several histone lysine demethylases [17]. Bruik’s team in 2007 identified JMJD6 as the first arginine demethylase that demethylates histone 3 and histone 4, which was confirmed only recently by Rosenfeld’s lab showing that JMJD6 is also able to demethylate snRNA [22], and by our lab identifying a non-histone target for JMJD6. Indeed, JMJD6 regulates oestrogen non-genomic signaling by demethylating ERα [23]. JMJD6 has also been reported to be a lysyl-5-hydroxylase on the splicing factor U2AF65 [20] and histone H3 and H4 [19], indicating that JMJD6 displays dual enzymatic properties.

In this paper, we have investigated the role of JMJD6 in breast tumourigenesis. Our *in vitro* and *in vivo* results clearly show that JMJD6 possesses anti-tumoural properties. Indeed, knock-down of JMJD6 increased several features of breast cancer including proliferation, migration, colony formation and tumour engraftment. These properties were confirmed by JMJD6 overexpression that showed the opposite effects. Of note, the catalytically dead mutant of JMJD6 loses its effects on the different processes establishing that JMJD6 enzymatic activity is involved in its anti-tumoural properties. As the mutated aminoacids are those required for Fe (II) binding, a prerequisite for both enzymatic activities [17,20], we cannot distinguish which activity is required for the observed effects. Specific inhibitors for each enzymatic activity would be helpful to clarify this point. However, our results are contradictory with those published in Lee et al [26], where they found that knock-down of JMJD6 in breast cancer cells decreases cell proliferation, migration and invasion. These discrepancies could be explained by the origin of the MCF-7 cells. In their paper, the authors found that JMJD6 is faintly expressed in MCF-7 cells compared to other cell lines. In our hands, JMJD6 expression was equivalent in the different breast cell lines (Fig 2A). Differences in the technical approach could also explain the discrepancies. We used stable cell lines infected with shRNA whereas Lee et al used siRNAs. In our hands, siRNAs had no effect on cell proliferation and cell migration (data not shown). We have also observed that siRNAs targeting JMJD6 had no effect on global arginine methylation (data not shown) whereas stable invalidation triggers an increase in global arginine dimethylation [23], suggesting that the deregulation of arginine methylation could be involved in the observed effects. This is supported by the role of arginine methylation on tumourigenesis. Indeed, upregulation of several PRMTs has been observed in various human cancers including breast cancers [9]. Moreover, the process of methylation on arginine residues has been linked to tumoural development. For example, methylation of the tumour suppressor protein programmed cell death protein 4 (PDC4) by PRMT5 was involved in breast tumourigenesis [14]. Methylation of ERα by PRMT1 associated with oestrogen non-genomic signaling activation is increased in aggressive breast cancer [15].

JMJD6 expression in 133 breast tumour samples revealed that a high expression of JMJD6 correlated with deleterious factors: younger patients, more pre-menopausal women, patients with higher SBR grade and more non luminal A tumours and could therefore be marker of bad prognosis. Moreover, high expression of JMJD6 was here associated with worse DFS. These results are in accordance with those from Lee et al that associated patient outcome with JMJD6 mRNA expression [26]. Its poor prognosis value has also been shown in lung adenocarcinoma [27]. Since we did not have another dataset for validation on TMA, we proposed to use bootstrap techniques in order to evaluate the sensitivity and robustness of DFS results. One thousand bootstrap samples of 133 observations were created by taking samples from the original dataset (n = 133) using random sampling with replacement. Means hazard ratio (HR) from bootstrap samples and associated 95% confidence intervals (95% CI) calculated are consistent with previous results from the original dataset that revealed an association between high JMJD6 expression and poor DFS. According to the bootstrap analysis (S3 Table), results obtained on original data and presented in the paper appear to be stable and strong.

In order to strengthen these results, an external dataset of mRNA expression (GSE1456-GPL96) among breast cancer patients was extracted from the PrognoScan plateform (http://www.abren.net/PrognoScan). Results about the prognostic value of JMJD6 mRNA expression on OS (S2 Fig) and DFS (S3 Fig) were similar to those obtain on TMA comforting that JMJD6 may be a marker of bad prognosis.

However, the results of survival analyses were not in accordance with our *in vitro* data. The conclusions from our *in vitro* data are not questionable as they are supported by concordant results obtained with overexpression and knock-down experiments. However, the analysis of JMJD6 expression in breast tumour specimens does not give any information about its activity. We could hypothesise that in breast tumours, JMJD6 could lose its enzymatic activity for several reasons. JMJD6 could be inactivated by interaction with inhibitory partners or by unidentified post-translational modifications. Alternatively, it is possible that JMJD6 could also be mutated in some tumours. Indeed, a mutation of JMJD6 on H187 a site known to be involved in Fe (II) binding has already been described (COSMIC database). Unfortunately the lack of tools to analyse JMJD6’s substrates prevents the verification of its enzymatic activity. Interestingly, similar results have been described for the LKB1 protein. The low expression of this tumour suppressor gene *in vitro* is associated with oncogenic features; however its high expression in the cytoplasm of breast tumours is associated with a decreased DFS probably because its antitumoral properties are lost when it is trapped with methylated ERα [28].

To conclude, by using a combination of different *in vitro* approaches, we demonstrated that the arginine demethylase JMJD6 possesses anti-tumoural properties. However, the study of JMJD6 expression in a cohort of breast tumours indicated that JMJD6 may instead be a marker of poor prognosis. Further work will be necessary to determine whether JMJD6 activity could be deregulated in breast cancer.

## Supporting Information

S1 Fig Kaplan Meier estimates of OS according to JMJD6 expression groups. (TIF)

S2 Fig Kaplan Meier estimates of OS according to JMJD6 mRNA expression. (TIF)

S3 Fig Kaplan Meier estimates of DFS according to JMJD6 mRNA expression. (TIF)

S1 Table Sample description: Distribution of clinical parameters. Clinical parameters (age at diagnosis, tumor size, menopausal status, lymph node involvement, SBR grading and hormonal expression) were described for the 133 patients included in the TMA study.
(DOCX)

S2 Table Distribution of JMJD6 expression. This table shows the distribution of JMJD6 expression in the 133 breast tumors. The results are presented according to the intensity of the staining and according to the percent of stained tumoural cells.
(DOCX)

S3 Table Internal validation of the JMJD6 prognostic effect on DFS by bootstrap method. This table shows results obtained by bootstrap method, with regard to the original parameters estimates. Means hazard ratio (HR) from bootstrap samples and associated 95% confidence intervals (95% CI) are presented with also the percentage of models where JMJD6 remained a prognostic factor of DFS among the 1000 models built.
(DOCX)
