# Supplementary figures and images for: Role of JMJD6 in Breast Tumourigenesis
Source: PLoS One. 2015 May 7;10(5):e0126181. doi: 10.1371/journal.pone.0126181 (PMC4423888; doi:10.1371/journal.pone.0126181)

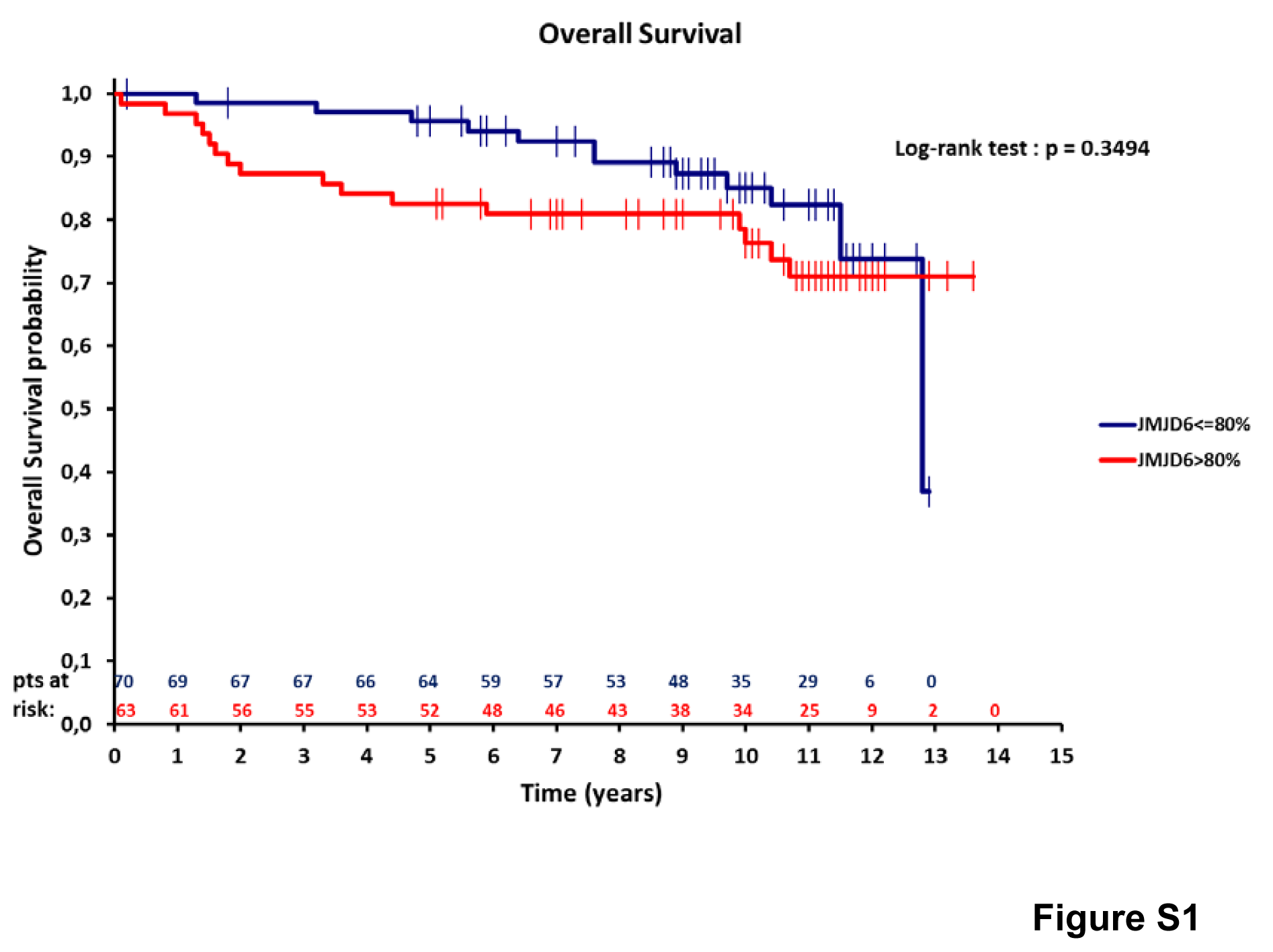

Supplement: S1 Fig — (TIF) [file pone.0126181.s001.tif]

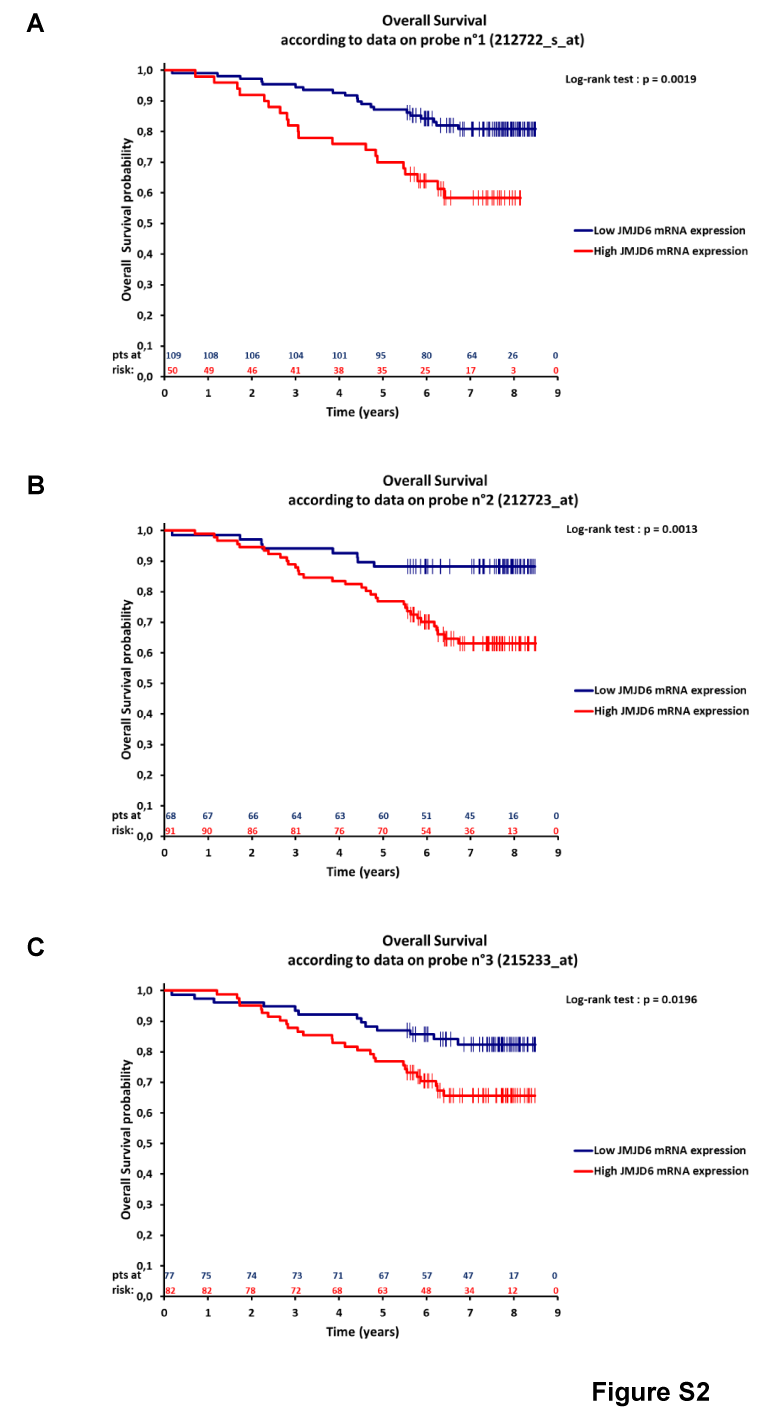

Supplement: S2 Fig — (TIF) [file pone.0126181.s002.tif]

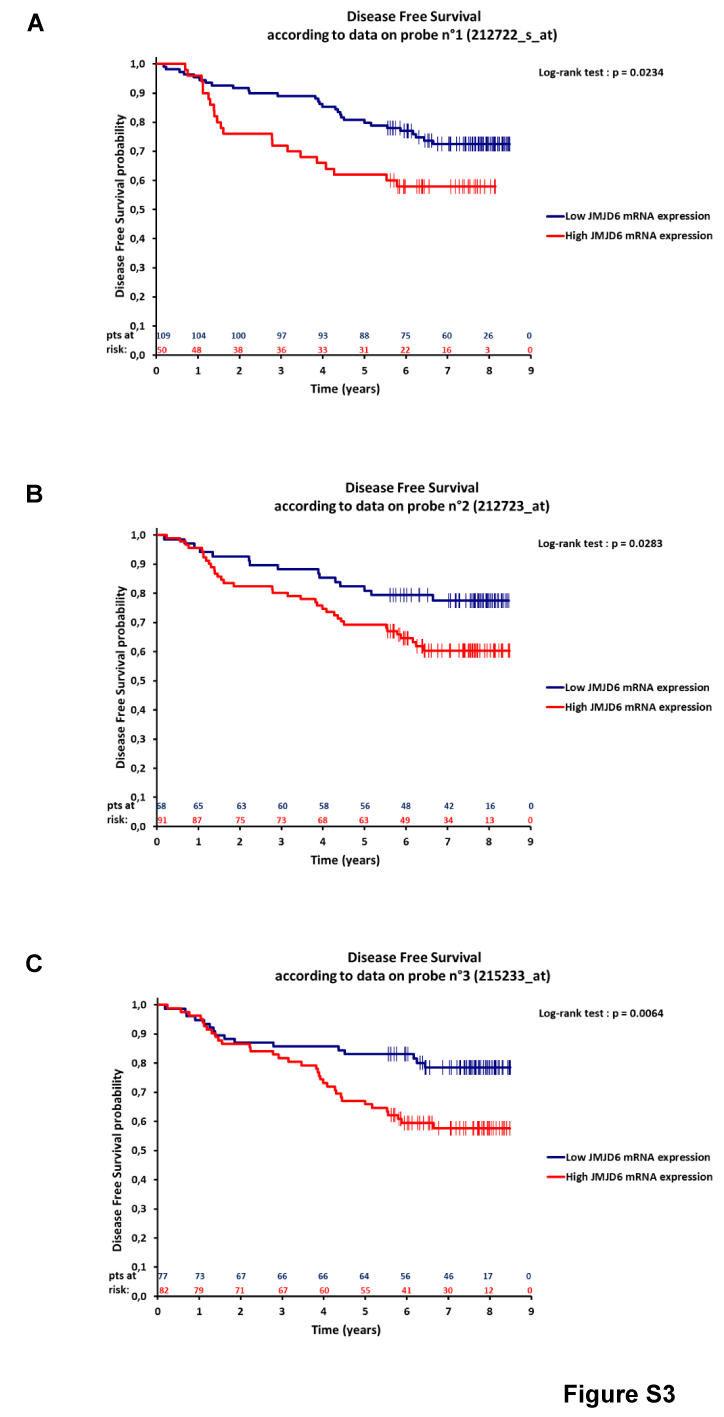

Supplement: S3 Fig — (TIF) [file pone.0126181.s003.tif]

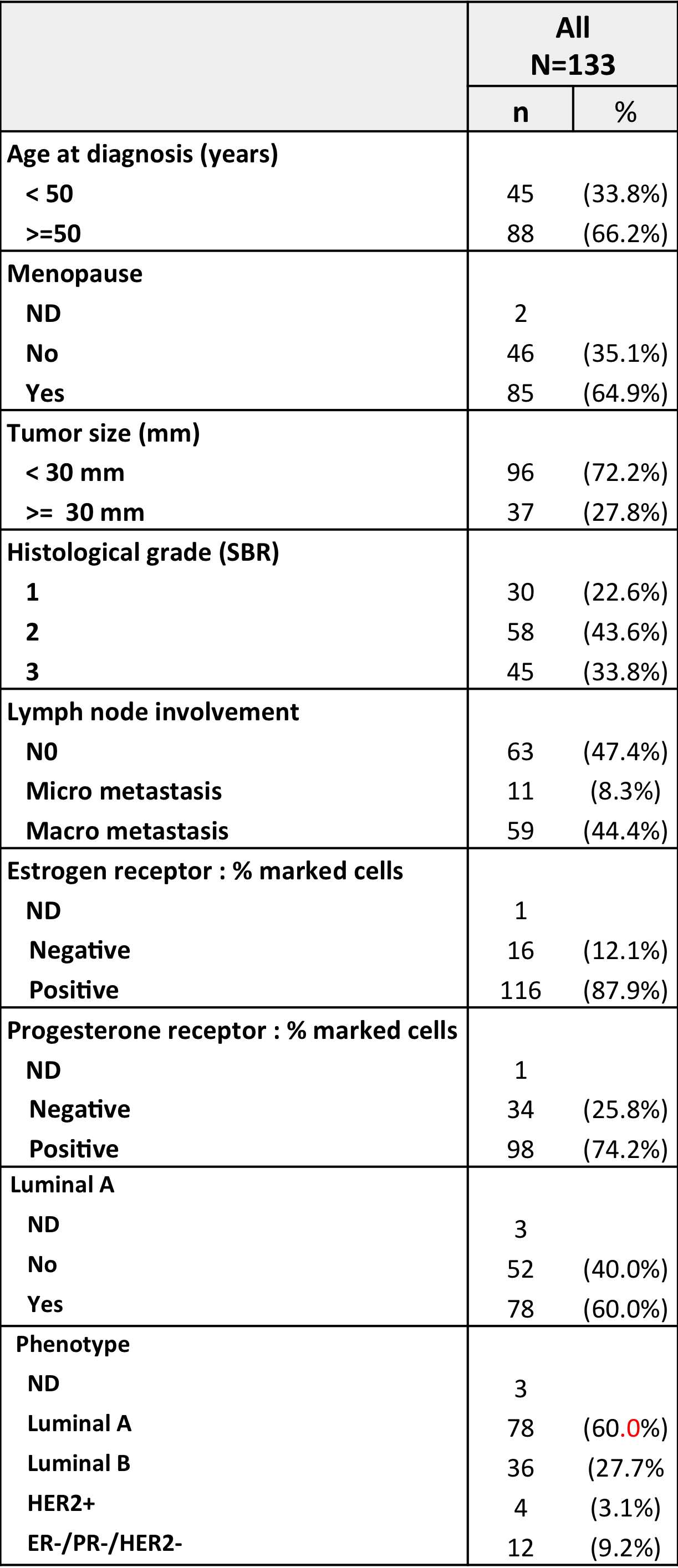


**Table S1: Sample description: Distribution of clinical parameters.**

Supplement: S1 Table — Clinical parameters (age at diagnosis, tumor size, menopausal status, lymph node involvement, SBR grading and hormonal expression) were described for the 133 patients included in the TMA study. (DOCX) [file pone.0126181.s004.docx]
