# Supplementary material for: Role of JMJD6 in Breast Tumourigenesis
Source: PLoS One. 2015 May 7;10(5):e0126181. doi: 10.1371/journal.pone.0126181 (PMC4423888; doi:10.1371/journal.pone.0126181)
Supplement: S2 Table — This table shows the distribution of JMJD6 expression in the 133 breast tumors. The results are presented according to the intensity of the staining and according to the percent of stained tumoural cells. (DOCX) [file pone.0126181.s005.docx]

**Table S2. Distribution of JMJD6 expression.**
