# Supplementary material for: Role of JMJD6 in Breast Tumourigenesis
Source: PLoS One. 2015 May 7;10(5):e0126181. doi: 10.1371/journal.pone.0126181 (PMC4423888; doi:10.1371/journal.pone.0126181)
Supplement: S3 Table — This table shows results obtained by bootstrap method, with regard to the original parameters estimates. Means hazard ratio (HR) from bootstrap samples and associated 95% confidence intervals (95% CI) are presented with also the percentage of models where JMJD6 remained a prognostic factor of DFS among the 1000 models built. (DOCX) [file pone.0126181.s006.docx]

**Table S3: Internal validation of the JMJD6 prognostic effect on DFS by bootstrap method.**
